# PREVALENCE OF NEUTRALIZING ANTIBODIES AGAINST POLIOVIRUS 1, 2, AND 3 IN HEALTHCARE PROFESSIONALS AGED 20-50 YEARS

**DOI:** 10.1590/1984-0462/2021/39/2019354

**Published:** 2021-02-03

**Authors:** José Cassio de Moraes, Maria Josefa Penon Rujula, Marcelo Otsuka

**Affiliations:** a Medical School of Santa Casa de São Paulo, São Paulo, SP, Brazil.; b Darcy Vargas Children’s Hospital, Secretaria de Estado de Saúde, São Paulo, SP, Brazil.

**Keywords:** Poliomyelitis, Antibodies, Health professionals., Poliomielite, Anticorpos, Profissionais de saúde

## Abstract

**Objective:**

To describe the prevalence of neutralizing antibodies against poliovirus (PV1, PV2, and PV3) in blood samples of healthcare professionals aged 20 to 50 years.

**Methods:**

Health professionals who serve children at Darcy Vargas Children’s Hospital and the Department of Pediatrics of Irmandade da Santa Casa de São Paulo. The sample size was calculated at 323 participants. The Mantel-Haenszel chi-square was used to verify differences between groups. The neutralization reaction detected human poliovirus antibodies. For susceptible individuals, vaccination with the inactivated+triple acellular polio vaccine was performed, and neutralizing antibodies were re-dosed after one week.

**Results:**

333 professionals were studied - 92.8% were immune to poliovirus 1, 86.5% to poliovirus 2, and 63.3% to poliovirus 3; 37% had titers less than 1:8 for any serotype, 5;1% had titers below 1:8 for all three. Vaccination with inactivated polio vaccine was performed for susceptible participants, and neutralizing antibodies were dosed after one week, showing increased titers for all polioviruses.

**Conclusions:**

Despite the detection of a significant percentage of individuals with low poliovirus antibody titer, the challenge with vaccination demonstrated immune response compatible with poliovirus immunity.

## RESUMO

**Objetivo:**

Descrever a prevalência de anticorpos neutralizantes contra poliovírus (tipos 1, 2 e 3) em amostra de sangue de profissionais de saúde com idade de 20 a 50 anos.

**Métodos:**

Profissionais de saúde que atendem crianças do Hospital Infantil Darcy Vargas e do Departamento de Pediatria da Irmandade da Santa Casa de São Paulo. O tamanho da amostra foi de 323 participantes. Os anticorpos contra poliovírus humanos foram detectados pela reação de neutralização. Para os indivíduos suscetíveis, foram administradas vacina para poliomielite inativada+tríplice e nova dosagem de anticorpos neutralizantes após uma semana. Utilizou-se o teste do qui-quadrado de Mantel-Haenszel para verificar as diferenças entre os grupos.

**Resultados:**

Foram estudados 333 profissionais - 92,8% eram imunes ao poliovírus 1; 86,5%, ao poliovírus 2; 63,57%, ao poliovírus 3; 37% apresentaram títulos inferiores a 1:8 para qualquer sorotipo; 5,1% tinham títulos abaixo de 1:8 para os três. Após a vacinação dos suscetíveis, houve elevação dos títulos para todos os poliovírus.

**Conclusões:**

Apesar da detecção de percentual significativo de indivíduos com baixo título de anticorpos para poliovírus, o desafio da vacinação demonstrou resposta imune robusta compatível.

## INTRODUCTION

According to the World Health Organization (WHO), in 2019, 125 cases of wild poliovirus (WPV) type 1 were reported in Afghanistan (24) and Pakistan (101).^1^ Even though most cases of paralysis due to WPV and circulating vaccine-derived polioviruses (cVDPV), diagnosed in children under five, polio can affect susceptible people of any age.^2^ ^,^ ^3^ ^,^ ^4^ Infection with WPV or cVDPV in countries certified as polio-free is a risk due to the increased flow of travelers through tourism, work, cultural exchanges, or political, religious, and cultural missions.^4^ Although unvaccinated children are the main group involved in the polio transmission chain, the role of adults with asymptomatic poliovirus infection should not be underestimated.

In Brazil, there is little information on immunity to polioviruses in adults, and we have not found any study on health professionals.^5^ We are also unaware of the percentage of health professionals vaccinated against polio. Bearing in mind that WPVs stopped circulating in the country three decades ago (the last record was in 1989), that many adults, especially those born before 1980, should not have been vaccinated against polio in childhood, and that there is a progressive reduction in the antibody titers against poliovirus, we consider it essential to research serum immunity in health professionals to assess the need for revaccination.

This study aimed to describe the prevalence of neutralizing antibodies against poliovirus - poliovirus 1 (PV1), 2 (PV2), and 3 (PV3) - in a sample of health professionals aged between 20 and 50 years old in the period from 2016 to 2017.

## METHOD

It is a cross-sectional study of health professionals (physicians and nursing professionals) who attended children at Darcy Vargas Children’s Hospital and the Pediatrics Department of Irmandade da Santa Casa de São Paulo during 2016 and 2017 and who agreed to take part in the study. The assumptions used to calculate the sample size were as follows: estimated prevalence of susceptible to one or more poliovirus = 30%,^6^ estimation error=5%, confidence level=95%, study power=80%, and losses=5%. The sample size was calculated in 323 participants. Professionals who had neutralizing antibody titers less than 1:8 were considered as potentially susceptible.^7^

Health professionals (physicians and nursing professionals) aged between 20 and 50 years who worked in the institutions involved in the care of children were included, excluding those who had received Oral Polio Vaccine (OPV) and/or Inactivated Polio Vaccine (IPV) in the past ten years. Based on the list of professionals from each of the categories in the two institutions involved, in order to select the participants, the draw was carried out using a simple casual sample of the subjects. If on the due day, the subject selected did not show up, he/she was replaced by another one from the same institution, of a similar age and in the same professional category. In 1980, to eliminate the disease, the Ministry of Health carried out two national vaccination campaigns in an indiscriminate manner for children under five years. We divided our population between those born before 1980 and those born after that year, who therefore had a chance to receive OPV in national campaigns.

To determine the seroprevalence of neutralizing antibodies, the blood samples collected were sent to the laboratory Fleury Medicina e Saúde. Serums were collected in appropriate tubes. The samples were kept at 4^o^C, in case the neutralization reaction was carried out within three days after collection, or frozen at -20^o^C, if the test was performed after three days. The Fleury laboratory transported the samples from the participating institutions to its central laboratory, in the neighborhood of Jabaquara, in São Paulo (SP), and provided a report containing the poliovirus results for each subject.

The neutralization reaction detected antibodies to human poliovirus. HEp-2 cells (which were used to culture the vaccine poliovirus and measure neutralization titers) were inoculated with standardized doses of PV1, PV2, and PV3, separately. Human serum was added to the cells at different dilutions, starting at 1:8 and doubling to 1:1024. After the 48-hour incubation period, the plates were read using an inverted microscope, looking for cytopathic effects. The last titer of the sample was considered the last dilution in which it was observed that the cytopathic effect was neutralized. The neutralization technique used was the one recommended by the WHO, with titrations below 1:8 considered negative for this methodology.^8^

Individuals who did not have neutralizing antibody titers for one or more polioviruses, for ethical reasons, were offered vaccination with diphtheria, tetanus, pertussis (acellular), and IPV (dTpa-VIP^R -^ Sanofi Pasteur) vaccine. After vaccinating individuals with low titers who agreed to participate, a new collection was carried out one week later. The same methods were used to evaluate seroconversion/change in neutralizing antibody titers.

In the statistical analysis, the prevalence of those potentially susceptible to each poliovirus was calculated. To compare prevalences, the Mantel-Haenszel chi-square test was used.

The research followed Conep’s recommendations, following Resolution No. 466, of December 12, 2012. The project was submitted to and approved by the Human Research Ethics Committee of Irmandade da Santa Casa de São Paulo, under number 379,176, of August 28, 2013. The study was conducted with the consent of health professionals by reading and signing the free and informed consent form.

## RESULTS

**Figure 1:**
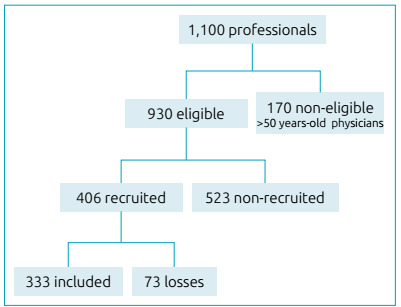
Flowchart for participant recruiting.

From 2013 to 2016, 333 out of the 1,100 health professionals from the two institutions were recruited, according to the flowchart shown in Figure 1. Out of the 333 professionals, 158 (47.4%) were physicians, and 175 (52.6%) were nursing personnel. As for the age group, 189 (56.8%) were 35 years old or younger, and 144 (43.2%) belonged to 36 or older.

Most health professionals, 209 (62.8% [95%CI 57.5-67.8]), had neutralizing antibody titers equal to or greater than 1:8 for the three polioviruses and were considered immune. The proportion of immune people did not vary according to the professional category (p=0.874; Table 1).

**Table 1:** Health professionals according to professional category and age in relation to immunity. São Paulo, 2015-2016.

	Professional category		Age		Total
	Nursing	Physician	<36 years old	≥36 years old	
Susceptible (n)	66	58	69	55	124
Immune (n)	109	100	120	89	209
Total (n)	175	158	189	144	333
% susceptible	37.7%	36.7%	36.5%	38.2%	37.2%
% lower limit	30.8%	29.5%	29.2%	30.5%	32.2%
% upper limit	45.1%	44.4%	43.6%	46.3%	42.5%

The proportion of immune people to the three polioviruses did not show a significant difference according to the age of the participants, but it was slightly lower for the youngest (p=0.743; Table 1).

Out of the 333 study participants, 24 (7.5% [4.8-10.4]) were susceptible to PV1, 33 (9.9% [7.0-13.5]) were susceptible to PV2, and 104 (31.2% [26.4-36.7]) were susceptible to serotype 3. Regarding poliovirus serotypes, 37.2% had titers below 1:8 for any serotype, and 5.1% had titers below 1:8 for all three.

The 124 professionals who had a low titer of neutralizing antibodies for one or more of the serotypes (37.2%) were offered, due to ethical reasons, IPV and participation in an immunological challenge. Out of these, 14 participated in the challenge, in whom a serum conversion of all individuals to the three polioviruses was observed.

## DISCUSSION

Protection against polio is fundamentally given by neutralizing antibodies against PV1, PV2, and PV3, considering that the protection is serotype-specific. The maintenance of high titers of poliovirus antibodies in adults can result from both vaccination and natural booster, exposure to WPV or vaccines.^9^

Although many believe that polio vaccines provide permanent immunity, several studies indicate that, over the years, there is a reduction in antibodies against polioviruses.^10^ ^,^ ^11^ ^,^ ^12^ ^,^ ^13^ ^,^ ^14^ ^,^ ^15^ ^,^ ^16^ ^,^ ^17^ ^,^ ^18^ In seroepidemiological surveys, it is rarely possible to identify among seronegative individuals how many have a primary or secondary failure in seroconversion. However, it is known that primary failures after using OPV are more frequent in countries with tropical climates, and not only the seroconversion rates but also the geometric mean of the antibody titers against poliovirus are lower than those observed after natural infection or vaccination with IPV.^10^

The lack of knowledge about the duration of immunization has determined that many countries use booster doses in adult populations. In Europe, several countries have introduced a booster dose of IPV, alone or combined with other vaccines (dT-IPV or dTpa-IPV), for adolescents, adults^19^ and, in 18 countries, for health professionals.^20^

In the United States, although polio revaccination has not been routinely indicated, since 2000, the Centers for Disease Control and Prevention recommend that adults who are not vaccinated or without proof of vaccination against polio receive three doses of IPV (zero, two, and six months) and that adults wishing to travel to endemic countries or recently reinfected by WPV, health professionals in contact with immunocompromised persons or working in laboratories with biological samples potentially contaminated with WPV, and adults in contact with children vaccinated with OPV receive a booster dose of IPV, even if they were vaccinated in childhood.^17^

Another factor that makes it difficult to analyze the duration of immunogenicity given by the vaccine concerns the group immunization observed with the OPV vaccine (currently, in Brazil, only for boosters). From the moment that many countries adopt IPV, due to the importance of polio triggered by the attenuated virus or its mutation,^1^ the booster effect of OPV is lost.

In our study, 62.8% of health professionals had titers greater than or equal to 1:8. The lowest percentage of seroimmune individuals was observed for serotype 3. We did not observe a significant difference between the two age groups and by gender. The comparison of these results with other studies (Table 1), which measured the presence of polio antibodies in adults, shows a situation similar to most countries concerning the prevalence of seronegative individuals, but worse than the Brazilian study carried out in 1967.^5^ The highest prevalence of seronegative individuals was observed, in most studies, for serotype 3, similarly to what was observed in our data. Few studies have been carried out on health professionals, making it difficult to compare.

**Chart 1:** Percentage of adults seronegative for poliovirus 1, 2, and 3, in different studies, according to country, year of publication, and number of adults assessed.

Country	Year	Number	PV1	PV2	PV3
Brazil^5^	1967	2,883	6	8	7
United States^5^	1967	3,202	16	15	25
United Kingdom^22^	1982	919 ≥ 15 years	11-23	12-15	8-24
United States^23^	1991	1,547	2	1	15
France^24^	1996	300	9	11	13
Italy^18^	1997	530	2	0.4	2
Australia^25^	1996-1999 (2005)	1,813	18	12	6
Oman (Arabia)^26^	2000	1,025	3	2	12
South Africa^10^	2001	776	6-8	6-8	13
Israel^27^	2001	521	1.4-1.5	0.5	2.5-10.8
Germany^11^	2007	2,046	2	2	7
Portugal^12^	2007	1,133	8	6	25
Uruguay^13^	2009	782	72	40	80
São Paulo^6^	2010	170	15	11	48
Germany^14^	2012	1,632	16,	9	18
Germany^15^	2012	424 (2004-2006) 427 (2008-2010)	16-15	9-11%	38-32
Italy^16^	2012	318	26	3	23
São Paulo*	2015	333	7	9	26

*this study; PV1: poliovirus 1; PV2: poliovirus 2; PV3: poliovirus 3.

The increase in the titers of neutralizing antibodies for all polioviruses within one week after vaccination of individuals who had low titers for one or more polioviruses (37% of the assessed subjects) demonstrated a rapid immune response in this population, suggesting the presence of previous immunity. It is believed that these individuals who had undetectable titers (less than 1:8) may have an immunological memory due to past exposure to the wild and/or vaccine virus, and that provocation could generate a quick and robust response. This rapid secondary response may be sufficient to prevent viral replication or paralytic disease. Individuals with immune memory, even in the absence of detectable circulating antibodies, can respond quickly enough to block the invasion of the Central Nervous System when exposed to the wild poliovirus or vaccine-derived virus.^19^ Still, it is necessary to consider that the poliovirus incubation period is quite short (ranging from one to six days).^20^ ^,^ ^21^ Therefore, the time to respond with high antibody titers in those who had previous exposure to polioviruses due to natural infection or vaccination may be insufficient to protect them against the disease.^22^

It should be noted that the chosen group was composed of health professionals with close contact with pediatric patients, in a period before the introduction of IPV, currently used in immunization in the 1st year of life, with the consequent possibility of herd immunization, which may determine bias in real prevalence of immunogenicity by polio vaccine over time. Research to assess the immune response in people seronegative for poliovirus is scarce and, as observed in the present study, they may have undetectable titers, but still be immune. The decrease in the use of OPV, on the other hand, may reduce herd immunization in the coming years, allowing, in the long run, a population more susceptible to poliovirus.

The response with the production of neutralizing antibodies observed in this study may suggest protection against polio over time, but with the concern, if this protection will be sufficient to prevent mainly neurological disease by polioviruses. It is up to the analysis of how the response to infection by cVDPV will be, which have been the biggest target of discussion of polio disease and reason for the current changes in the vaccine schedule, with the progressive withdrawal of OPV.

As limitations, it is noteworthy that an important proportion of drawn professionals refused to participate in the study or did not comply with the necessary procedures to be included. The vaccination status of the professionals was not subject to analysis with documented data since most of them did not have the vaccination record book used in their childhood. Oral information was based on the recall of parents or participants about vaccines received in childhood.

The challenge carried out with the inactivated vaccine to susceptible professionals had little adherence. Most of them, even though they knew about the results, refused to receive the inactivated vaccine offered or else did not show up on the 7^th^ day after vaccination for new blood collection.

In any case, measures for polio eradication worldwide must continue, allowing, after eradication, besides the benefits resulting from the elimination of the disease, cost reduction in the order of 40 to 50 billion dollars.
